# Epidemiology and Molecular Identification of *Coenurus cerebralis* in Sheep and Goats in Mpwapwa District, Tanzania: Factors to Consider in Control Plans

**DOI:** 10.1155/vmi/5055115

**Published:** 2024-12-05

**Authors:** Veneranda Philipo, Athumani Msalale Lupindu, Jahashi Saidi Nzalawahe

**Affiliations:** ^1^Department of Veterinary Medicine and Public Health, College of Veterinary Medicine and Biomedical Sciences, Sokoine University of Agriculture, P.O. Box 3021, Morogoro, Tanzania; ^2^Department of Academics, Livestock Training Agency, Mpwapwa Campus, P.O. Box 51, Mpwapwa, Dodoma, Tanzania; ^3^Department of Veterinary Microbiology, Parasitology and Biotechnology, College of Veterinary Medicine and Biomedical Sciences, Sokoine University of Agriculture, P.O. Box 3019, Morogoro, Tanzania

**Keywords:** abattoir postmortem survey, *Coenurus cerebralis*, cyst, Mpwapwa, small ruminants, *Taenia multiceps*, Tanzania

## Abstract

Cerebral coenurosis is a significant disease that affects sheep and goats worldwide. Studies conducted in northern and southern Tanzania have determined its magnitude and determinants. However, limited information from other regions of the country hinders the development of comprehensive national control plans. This study was conducted to determine the prevalence, knowledge, risk factors, and molecular identity of *Coenurus cerebralis* in sheep and goats in Mpwapwa District, Tanzania, in order to establish the preliminary status of the disease in the central regions. Data were collected through postmortem examinations of 84 sheep and 295 goat heads for cysts, polymerase chain reaction (PCR) and sequencing for species identification, and a structured questionnaire of 303 small ruminant keepers for knowledge and identification of risk factors. The overall prevalence of cerebral coenurosis in sheep and goats was 13.5% (95% confidence interval (CI) 10.3–17.2). In goats, the prevalence of cerebral coenurosis was comparatively higher 15.9% (95% CI 11.9–20.6) than in sheep 4.8% (95% CI 1.3–11.8) (*p* = 0.008). Multivariate logistic regression analysis identified the origin of sheep and goats (Chipogoro: odds ratio (OR) = 7.54, 95% CI 1.96–28.97, and Iwondo: OR = 3.90, 95% CI 1.04–14.61) as a risk factor. The average knowledge score among small ruminant keepers on disease detection was 60.7%, on disease infection cycle and control was 10.85%, and on zoonotic potential was 1.39%. Phylogenetic analysis of Cytochrome C oxidase subunit I (CO1) gene partial nucleotide sequences revealed the two distinct clusters of *Taenia multiceps*, one from Saudi Arabia in 2021 and another from Egypt in 2018, Peru in 2017, and China in 2016. These findings suggest cerebral coenurosis is locally widespread and highlight the importance of considering animal movement patterns, livestock keeper's knowledge, and good husbandry practices when planning for control measures of cerebral coenurosis.

## 1. Introduction

Cerebral coenurosis is an important disease that affects sheep and goats, causing significant economic losses [1–5]. It is caused by *Coenurus cerebralis* the larvae of *Taenia multiceps* parasite. Dogs serve as the parasite's definitive host, and small ruminants serve as its typical intermediate host during its life cycle. Other intermediate hosts include humans, cattle, pigs, horses, buffaloes, and yaks, while other definitive hosts include wild canids like foxes, coyotes, and jackals. *Taenia multiceps* life cycle involves eggs, larvae, and adult stages. It is named so because of the presence of multiple heads on the Coenurus wall. Morphologically, adult worms are whitish, dorsoventrally flattened, and segmented with a length ranging from 400 mm to 1000 mm and a width of 5 mm. Also, they have a scolex of 746–956 *μ*m diameter with four suckers and a rostellum equipped with small and large hooks (22 to 32) arranged into crowns [6]. The lengths of the small and large hooks range from 98 and 136 *μ*m and 157 and 177 *μ*m, respectively [6]. Mature *Coenurus cerebralis* appears as a cystic fluid-filled sac (bladder-like) with a size of 2–5 cm in diameter. It presents protoscolices which are grossly seen as whitish spots in a clear/white fluid visible on the transparent (thin) cyst wall and germination membrane. These protoscolices are multiple small, developing head regions of the tapeworm which are capable of developing into adult tapeworms if they are ingested by the definitive host. They contain four suckers, a large rostellum, and small hooks arranged in two rows. Cerebral coenurosis disease is of public health concern since humans are also affected. Although it is a rare zoonosis, some cases in humans have been reported in North America, South America, Israel, Italy, and Egypt [7–10]. *Coenurus cerebralis* is located in the intermediate hosts' spinal cord and brain. Definitive hosts like dogs get infected when they consume the spinal cord or brain, which contain *Coenurus cerebralis* cysts. The cysts develop into *Taenia multiceps*, which produce proglottids containing eggs that contaminate the environment, including water sources and grazing areas. Small ruminants and other intermediate hosts get infected when they consume eggs of the parasite from water and pastures [11]. Clinical signs in intermediate hosts can manifest in acute or chronic stages, with majority being neurological signs like ataxia, circling, hind leg paralysis, and slight head aversion. These neurological signs result to a serious drop in production, mortalities, and economic losses [1–5, 12, 13]. Pathological findings, imaging techniques like (ultrasonography, magnetic resonance imaging, and computerized tomography), and molecular tests are used to make diagnoses of the disease. Gross pathological features are considered to be pathognomonic and present protoscolices which are seen as white spots in a clear/white fluid visible on the transparent cyst wall and germination membrane. The recommended treatment for cerebral coenurosis in animals is surgical cyst removal although it is often not carried out because of the limited finances of many sheep and goat owners and less experience of doing the procedure in animals [14]. Also, minimum capacity and infrastructures hinder the procedure, especially in developing countries. In definitive hosts like dogs, the infection is normally subclinical, although, in heavy infestations, nonspecific digestive system signs may occur. To break the cycle, the disease should be controlled in dogs by using praziquantel drug routinely. Cerebral coenurosis cases have been reported worldwide, with the majority of cases occurring in African countries [1–5, 12, 13, 15–24]. In Tanzania, different studies have been conducted in the northern and southern regions to quantify the disease magnitude, losses, and determinants [5, 12, 18, 21–24]. However, disease profiles in other regions of Tanzania remain unknown. Therefore, the present study aimed to determine the prevalence, associated risk factors, and molecular identity of *Coenurus cerebralis* in sheep and goats in Mpwapwa District, Tanzania. Also, the study assessed the level of awareness, local knowledge, and disease management practices. The purpose was to generate a baseline information that will facilitate the development of effective policies, strategies, and guidelines for controlling the disease.

## 2. Materials and Methods

### 2.1. Study Area

**Figure 1:**
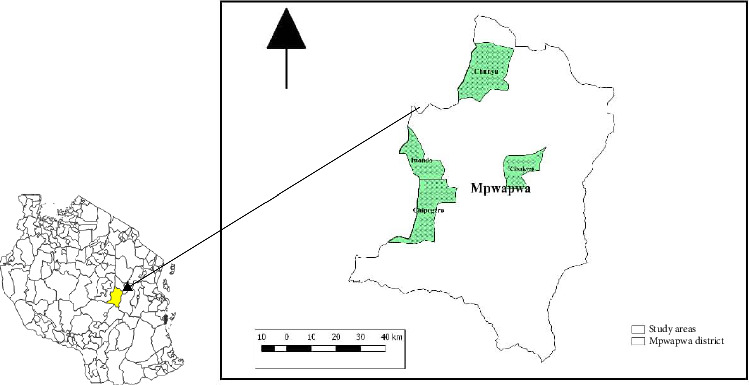
Map of Mpwapwa District, showing the study areas. The insert is a map of Tanzania.

The study was done in Mpwapwa District, which is located at the central part of Dodoma Region, Tanzania (Figure 1). The district has a human population of 403,247 [25]. It is bordered by Kongwa District to the north; Kilolo District to the south; Kilosa and Gairo districts to the east; and Chamwino District to the west. The district lies between latitude 6.567° South of the Equator and longitude 36.600° East of Greenwich. It covers an area of 7489 km^2^ and has four administrative divisions, namely, Mpwapwa, Mima, Kibakwe, and Rudi. It also has 33 wards and 113 villages. Most parts of the district receive an average of 712 mm of annual rainfall. The temperature in the district ranges between 20.2°C and 29.7°C. The estimated population of sheep, goats, and dogs is 86,047, 302,561, and 13,485, respectively [26].

### 2.2. Study Design

A cross-sectional survey was conducted from November 2023 to March 2024 that included postmortem survey for cerebral coenurosis in slaughtered small ruminants, molecular identification of *Coenurus cerebralis*, and a household questionnaire survey.

### 2.3. Sample Size Determination

The sample size was calculated by using the formula: *n* = *Z*^2^PQ/*D*^2^ [27], where *n* = sample size; *Z* = standard normal deviation, set at 1.96 corresponding to a 95% confidence level; *Q* = (1 − *P*); *D* = allowable error (degree of accuracy), set at *p* value < 0.05, which is 5%; and *P* = known or estimated prevalence from the target population. The estimated prevalence of 44.4% was used for the postmortem examination survey study of cerebral coenurosis in small ruminants based on the study conducted in the Ngorongoro District [18]. The estimated proportion of 27% was used for the questionnaire survey study, as an overall proportion of households feeding offal to their dogs in a study conducted in northern Tanzania [22]. Therefore, the sample size used in small ruminants was 379, i.e., 1.96^2^ × 0.444 × 0.556/0.0025 = 379.34, ∼379.

The proportion of sheep or goats was calculated using the population of sheep or goats at the district divided by the total population of both sheep and goats at the district, multiplying by the sample size. Therefore, the number of sheep sampled was 84, i.e., (86, 047/388, 608∗379) = 83.9∼84, and the number of goats sampled was 295, i.e., (302, 561/388, 608∗379) = 295.08∼295. The sample size used for households in the survey of questionnaires was 303, i.e., 1.96^2^ × 0.27 × 0.73/0.0025 = 302.87∼303. The number of representative households in each ward was obtained based on the proportion of the total number of households owning livestock in each ward:

(1) $$\begin{array}{c} i.e.,\text{ Number of representative households}=\frac{\text{Total households in }a\text{ ward}\times \text{sample size}}{\text{Total households in all selected wards}}. \end{array}$$

Therefore,

(2) $$\begin{array}{c} \text{Number of representative households in Chunyu ward}=\frac{3524}{12335}\times 303=86.5∼87,\\ \text{Number of representative households in Iwondo ward}=\frac{2345}{12335}\times 303=57.6∼58,\\ \text{Number of representative households in Kibakwe ward}=\frac{3815}{12335}\times 303=93.71∼93,\\ \text{Number of representative households in Chipogoro ward}=\frac{2651}{12335}\times 303=65.1∼65. \end{array}$$

### 2.4. Ethical Consideration, Sampling Technique, Data Collection, and Processing

#### 2.4.1. Ethical Consideration

Permission to conduct this research was obtained from the Ethical Review Committee of Sokoine University of Agriculture with reference number SUA/DPRTC/R/186. Permission to collect data was obtained from the President's Office, Regional Administration, and Local Government Authorities. Verbal consents were obtained from participants before data collection, and the confidentiality of information was adhered.

#### 2.4.2. Sampling

The district possesses nine working primary livestock markets which are Ilolo, Chogola, Gulwe, Msagali, Mima, Kibakwe, Namba Thelathini, Rudi, and Chipogoro. At the markets, sheep and goats are sold and slaughtered for consumption within the district and some are sold and transported to secondary livestock markets, cross-border livestock markets, slaughterhouses that do meat processing business, feedlots, and ranches. A single visit was paid to each livestock market during the study period in compliance with the district's yearly livestock market schedule. Only animals brought for slaughter were included in the study. Since Ilolo livestock market slaughter an average of 60 to 70 small ruminants per market day, 25% of the animals brought, i.e., 15 to 18 sheep or goats were selected using a systematic random sampling technique where every fourth animal was selected for *Coenurus cerebralis* cyst examination without considering the health status of animals. For the remained livestock markets, which slaughter an average of 5 to 10 animals per market day, 50% of the animals brought, i.e., 3 to 5 sheep or goats were selected using a systematic random sampling technique in which every second animal was selected for *Coenurus cerebralis* cyst examination without considering the health status of animals. In these markets, 50% of brought animals were selected because the number of slaughtered animals at the markets is usually low due to a minimum number of customers influenced by market locations in remote areas. This is contrary to Ilolo market which is located at an urban setting where customers of slaughtered meat are plenty. Selected animals were marked using a permanent marker and individually identified by age which was assessed by dentition, i.e., through observing the replacement of incisors teeth. A one-year-old was the one with two permanent teeth, 2 years old with four permanent teeth, 3 years old, with six permanent teeth, 4 years old with eight permanent teeth, and 5 years old with weared, teared, and loosened teeth. Also, sex, breed, and place of origin were identified. A total of 84 sheep and 295 goats were randomly sampled from the livestock markets during the study period. Additionally, a purposive sampling technique was used to select four representative wards among 33 wards of the four divisions of the district, for the questionnaire survey study. The questionnaire was designed to assess risk factors and small ruminant keeper's knowledge on cerebral coenurosis. Selected wards were Chunyu, Iwondo, Kibakwe, and Chipogoro. The wards were selected because they frequently reported cases of the disease and possessed larger populations of sheep and goats [26], hence contributing significantly to the number of animals brought to livestock markets for sale or slaughter. A structured questionnaire that combined closed and open-ended questions was administered to 303 small ruminant keepers by the Principal investigator (V. P) and two animal health experts (F. C and A. M). Participants were selected using a simple random technique that employed a random number generator method to identify households from the wards.

#### 2.4.3. Postmortem Survey of Cerebral Coenurosis in Slaughtered Small Ruminants

Ante-mortem examination was conducted by a qualified veterinarian V. P (Principal investigator) before the postmortem examination. The procedure followed standard ante-mortem inspection procedures as described by [28]. Records of animals presenting neurological signs were documented. The postmortem procedure was performed according to the method described by [29]. The heads of slaughtered animals were separated from the rest of the carcass, and the skull was removed by ventral disarticulation of the atlanto-occipital joint. Thereafter, the head skins were removed, and a machete was used to make two parallel incisions on the parietal bone and a cross-sectional cut in the region immediately caudal to the frontal bone. After that, the meninges were cut to reveal the brain tissue, and the bones were extracted. The animal's entire brain was then inspected for gross pathological lesions. Using a sharp knife, a longitudinal incision was made along the animal's back to reveal the spinal column in order to examine the spinal cord. The spinal cord was made visible by carefully removing the bone and surrounding tissues from the vertebral column. The spinal cord was then inspected to check for cysts. Depending on the stage of development, the cysts obtained contained immature forms or mature protoscoleces, which were seen as white spots on the inner surface of the cysts. The obtained cyst number, size, and location were documented. Animals found with cyst(s) were considered positive for the disease. Those without cyst(s) were considered negative for the disease. The cyst(s) obtained were preserved in 70% ethanol.

#### 2.4.4. Molecular Identification of *Coenurus cerebralis*

##### 2.4.4.1. Deoxyribonucleic Acid (DNA) Extraction

Fifty-one cysts collected from postmortem examinations of sheep and goats were transported to the Biochemistry Research Laboratory of the Department of Veterinary Physiology, Biochemistry, and Pharmacology of Sokoine University of Agriculture for molecular analysis. The Quick-DNA Mini Prep Plus Kit (Zymo Research) was used to extract genomic DNA from solid tissues following the manufacturer's instructions. Less than or equal to 25 mg of both protoscoleces and cyst walls were used to extract genomic DNA. In summary, each tissue sample was placed in a microcentrifuge tube and treated with 100 *μ*L of water, 100 *μ*L of solid tissue buffer, and 20 *μ*L of Proteinase K. After carefully mixing the components, the mixture was incubated for 3 hours at 55°C to achieve complete tissue digestion. The mixtures were centrifuged at > 12,000 × g for 1 min to remove insoluble debris, and the aqueous supernatant was then transferred to clean microcentrifuge tubes. Each tube was filled with 400 *μ*L of genomic binding buffer, which was well mixed. After that, the mixtures were transferred into Zymo-SpinTMIIC-XLR Column collection tubes, and they were centrifuged for one minute at ≥ 12,000 × g. The collection tubes with the flow-through were disposed of. In new collection tubes, 400 *μ*L of DNA Pre-Wash Buffer was added to the spin column, and it was centrifuged at ≥ 12,000 × g for a minute. The flow was discarded. Then, 700 *μ*L of g-DNA Wash Buffer was added to the spin columns and centrifuged for a minute at ≥ 12,000 × g. The collection tubes were emptied. Then, 200 *μ*L of g-DNA wash buffer was added and centrifuged for 1 min. The flow-through collection tubes were disposed of. After that, the spin columns were transferred to clean microcentrifuge tubes, and 100 *μ*L of DNA elution buffer was added directly to the matrix. After 5 minutes of room-temperature incubation, the solutions were centrifuged for 1 minute at maximum speed in order to extract the DNA. After that, the extracted DNA was kept at ≤ −20°C.

##### 2.4.4.2. Cytochrome C Oxidase Subunit I (COI) Amplification

The fragments of COI of 450-bp length from mitochondrial DNA (mtDNA) were amplified based on standard operating procedures, as was done by previously published methods created for molecular speciation of Taenia infection by [30, 31]. The CO1 (450 bp) primers used were JB3 (5′-TTTTTTGGGCATCCTGAGGTTTAT-3′) and JB4.5 (5′-TAAAGAAAGAACATAATGAAAATG-3′). Polymerase chain reaction (PCR) amplification was conducted in a 25-*μ*L reaction volume containing 2 *μ*L of extracted DNA, 5 *μ*L of FIREPol Master Mix Ready to Load with 7.5 Mm MgCl_2_ from Solis Biodyne (Teaduspargi 9, 50411 Tartu, Estonia), 1 *μ*L of forward primer (JB3), 1 *μ*L of reverse primer (JB4.5), and 16 *μ*L of nuclease-free water. The cycling conditions consisted of 5 minutes of initial denaturation at 95°C, followed by 35 cycles of denaturation at 95°C for 30 s, then, 30 s of annealing at 52°C, and 1 minute of extension at 72°C. The final extension was performed to complete the elongation of the PCR products at 72°C for 5 min. The products from PCR were then separated by gel electrophoresis, where agarose gel of 1.0% strength was prepared by dissolving 1.0 g of agarose powder into 100 mL of IX sodium borate buffer in a conical flask and heated on a hot plate until dissolved completely and stained with 80 *μ*L of GreenStar Nucleic Acid Staining Solution 1 (Bioneer Corporation). 3 *μ*L of each sample was added to each gel well, and one well was loaded with 3 *μ*L of a 100 bp DNA ladder to indicate the size of any fragments. Electrophoresis was allowed to run for 60 min using a voltage of 100 V. The gel was transferred to the gel documentation machine (Gel Doc EZ Imager from Bio-Rad Laboratories) for visualization.

##### 2.4.4.3. Sequencing for Genotyping

Twelve amplicons (Table 1) selected from samples with good PCR amplification were sent to Macrogen Europe (Meibergdreef 57, 1105 BA, Amsterdam, the Netherlands) for sequencing. The PCR products were purified and sequenced directly using the BigDye Terminator Cycle Sequencing Kit (Applied Biosystems, Foster City, CA, United States of America) and a genetic analyzer (ABI 3730xl System from Applied Biosystems). Geneious Prime (version 2024.0.5) software was used to clean and assemble the raw sequence data to obtain consensus sequences.

#### 2.4.5. Household Questionnaire Survey

Selected households from Chunyu, Iwondo, Kibakwe, and Chipogoro wards were visited and interviewed. A structured questionnaire was used to assess small ruminant keepers' knowledge about disease detection (definitions, hosts, signs, diagnosis (both clinical and postmortem)), disease infection cycle (etiology and transmission), and disease control practices. It also identified risk factors for cerebral coenurosis, including the origin of small ruminants, grazing systems, water sources, and deworming practices. The risk factors were selected based on the disease ecology, i.e., origin of small ruminants aimed to investigate differences in the care of these animals or possible exposure to factors prior to the current farm situation. Grazing systems aimed to assess variations in the likelihood to influence animal exposure/contact with pastures contaminated with dog feces containing *Taenia multiceps* eggs from different grazing systems. Likewise, water sources were studied to investigate the differences in transmission of *Coenurus cerebralis* parasites from different water sources contaminated with dog feces containing *Taenia multiceps* eggs. Also, deworming practices were studied to assess differences in the disease burden between dewormed and nondewormed animals. Data were collected through face-to-face interviews and recorded using the EpiCollect 5 application (version 7.0.3). The selection criteria for household interviewees depended on the ownership of small ruminants and the ownership or non-ownership of dogs, as well as the interviewee's convenience. A total of 303 households were interviewed.

### 2.5. Data Analysis

The data were entered in Microsoft Office Excel and then cleaned, coded, and analyzed using STATA 14 software. Descriptive statistics like means and frequencies were computed. The proportion of sampled animals that tested positive for cerebral coenurosis based on postmortem and PCR findings was used to calculate the disease prevalence. The Chi-squared test was employed to assess the statistical differences between proportions.

For knowledge assessment, there were 15 questions and each was assigned 1 mark for correct answer and 0 for incorrect answer. A summated index scale was used to categorize small ruminant keepers who participated in the questionnaire survey study into those with no knowledge about cerebral coenurosis (0), those with low knowledge about cerebral coenurosis (1 to 5 points), those with moderate knowledge about cerebral coenurosis (6 to 10 points), and those with high knowledge about cerebral coenurosis (11 to 15 points). The overall average scores from all participants with respect to each aspect of cerebral coenurosis were determined.

Factors associated with cerebral coenurosis were analyzed by using logistic regression in STATA 14 software to assess the relationship between cerebral coenurosis (yes or no) as the dependent variable and household practices related to sheep and goats as independent variables. The independent variables selected were the origin of sheep and goats, tap water source, seasonal holes and canals water source, communal grazing, cut and carry, deworming, and dog ownership. Univariate analysis was conducted before multivariate analysis. Variables with a *p* value of less or equal to 0.25 in the univariate analysis qualified for inclusion in the multivariate analysis. Multivariate analysis was done using a backward stepwise approach where all selected independent variables were entered into the model at once. The most nonsignificant independent variables were dropped one after another iteratively, retaining only those with a *p* value of less than 0.05 and the confounders. A factor was considered a confounder if its removal from the model caused a relative change of 25% or an absolute change of 0.1 in the coefficients of other variables. Interactions between variables in the final model were assessed. The likelihood ratio test was used to test the goodness of fit of the model at a significance level of 5%.

Phylogenetic analysis was done by subjecting nucleotide sequences to the Basic Local Alignment Search Tool (BLAST) to determine the identity of the study nucleotide sequences by comparing them with other published Taenia species available in the GenBank database. Using Clustal W in Molecular Evolutionary Genetic Analysis (MEGA) 7, the COI gene partial nucleotide sequences from this investigation were aligned with selected reference COI gene nucleotide sequences from GenBank. The phylogenetic analysis was performed using the maximum likelihood method, which utilizes the bootstrap test method with 1000 replicates, which is found in MEGA 7. The phylogenetic tree was constructed based on the partial nucleotide sequences of the *Taenia* species COI gene from 11 samples, which produced consensus sequences and four nucleotide sequences from reference strains obtained from GenBank.

## 3. Results

### 3.1. Flock Characteristics and Management of Brain Materials From Sheep and Goats

Out of the 303 animal keepers interviewed, 168 (55.45%) kept both sheep, goats, and dogs; 115 (37.95%) kept goats only; 17 (5.61%) kept sheep and goats only; and 3 (0.99%) kept sheep only. The average number of small ruminants (both sheep and goats) owned by livestock keepers was 14, with a minimum number of 1 and a maximum number of 203. The average number of dogs owned by animal keepers was 3, with a minimum number of 1 and a maximum number of 13. Out of 303 small ruminant keepers interviewed, 29.37% (89/303) reported encountering cases of cerebral coenurosis in sheep or goats in the past 12 months either clinically or through postmortem examinations. The cases were considered valid due to the reasonable knowledge of 60% on disease detection from small ruminant keepers identified from this study. Various management practices were applied after encountering these cases. Among the respondents, 44.94% (40/89) decided to sell the affected animals, while 20.22% (18/89) took no action. Additionally, 16.86% (15/89) chose to slaughter the animals for home consumption, 11.24% (10/89) applied heat to the heads of sick animals using hot iron, and 6.74% (6/89) treated them with antibiotics. There were variations in the practices for managing brain materials reported by small ruminant keepers who encountered cases of cerebral coenurosis in their livestock-keeping history and decided to slaughter affected animals. Among them, 56.5% (35/62) gave the brain materials to dogs, 40.3% (25/62) threw the brain away, 1.6% (1/62) consumed the brain, and an equal percentage 1.6% (1/62) threw it into toilet pits.

### 3.2. Prevalence of Cerebral Coenurosis, Clinical Signs, Cysts Location, Number, and Size in Sheep and Goats

**Figure 2:**
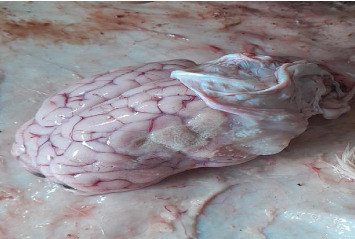
The brain of a sheep containing cysts.

**Figure 3:**
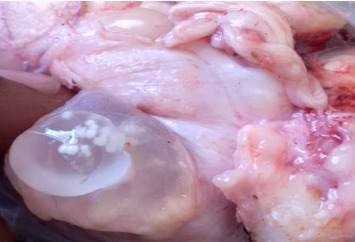
The brain of a goat containing a cyst.

**Figure 4:**
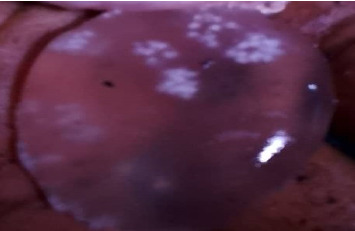
A cyst recovered from the brain of sheep.

**Figure 5:**
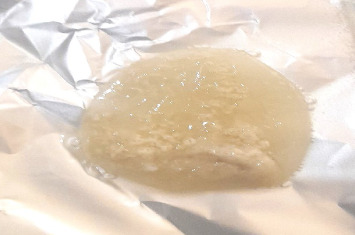
A cyst recovered from the brain of a goat.

The postmortem surveys of viable cysts (Figures 2, 3, 4, and 5) from small ruminants at slaughter facilities revealed a prevalence of 13.5% (95% CI 10.4%–17.3%) for cerebral coenurosis. The prevalence of the disease in sheep was 4.8% (95% CI 1.3%–11.8%), and the prevalence in goats was 15.9% (95% CI 11.9%–20.6%). The difference in prevalence between the two species was statistically significant (*X*^2^ = 7.0, *df* = 1, *p* = 0.008). This means that cerebral coenurosis was more prevalent in goats than in sheep. Out of the four divisions, Rudi division had the highest prevalence of cerebral coenurosis in both sheep and goats (Table 2). The prevalence of the disease across age groups was not statistically significant (Table 3). The location of the cysts in the brain of small ruminants showed that *Coenurus cerebralis* was most commonly found in the cerebral hemispheres (80.39%), while the remaining proportion (19.61%) had cysts in the cerebellum. Within the cerebral hemispheres, the left side contained 36.84% in goats and 33.33% in sheep, while the right side contained 63.16% in goats and 66.67% in sheep. The location of *Coenurus cerebralis* in the brain was statistically significant (*X*^2^ = 52, *df* = 3, *p* ≤ 0.001) (Table 4). This means that location of cyst in the right side of the brain was more common. From the total 51 positive cases, the number of cysts ranged from 1 to 3 cysts per infected animal, with one cyst in 37 (72.55%), two cysts in 11 (21.57%), and three cysts in 3 (5.88%). Of all the study animals, no cysts were recovered in the spinal cord. Only 19.6% (10/51) of animals found infected at postmortem presented neurological signs during ante-mortem inspection. The clinical signs presented by the study sheep and goats were a slight head aversion, incoordination, and circling. The most clinical sign was circling 4 (40%), followed by incoordination 3 (30%) and a slight head aversion 3 (30%). Cysts' sizes ranged from 2 to 6 cm. Clinical signs were detected in 90% (9/10) of animals found with cysts sized 4 to 6 cm and 10% (1/10) of animals with cysts sized 2 to 3 cm.

### 3.3. PCR Product Results From Gel Electrophoresis

**Figure 6:**
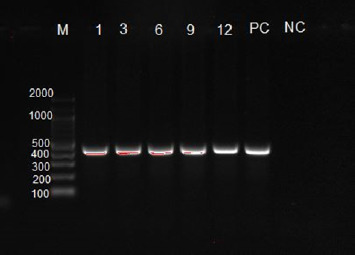
*Coenurus cerebralis* DNA fragments from gel electrophoresis with the size of 450 bp.

The DNA fragments from 51 samples were visualized on the gel documentation machine (Gel Doc EZ Imager from Bio-Rad Laboratories) and were observed as gray bands against a black background with a size of 450 bp (Figure 6).

### 3.4. Phylogenetic Analysis Results

The COI gene partial nucleotide sequences of Taenia species with 450 bp long from 11 samples produced consensus sequences. A BLAST for COI gene nucleotide sequences revealed a maximum sequence identity of 100% with *Taenia multiceps* species (LC271737) from Egypt, 99.51% with *Taenia multiceps* species (MZ346598) from Saudi Arabia, 99.00% with *Taenia multiceps* species (KX547633) from China, and 98.78% with *Taenia multiceps* species (KX511892) from Peru (Table 5)

**Figure 7:**
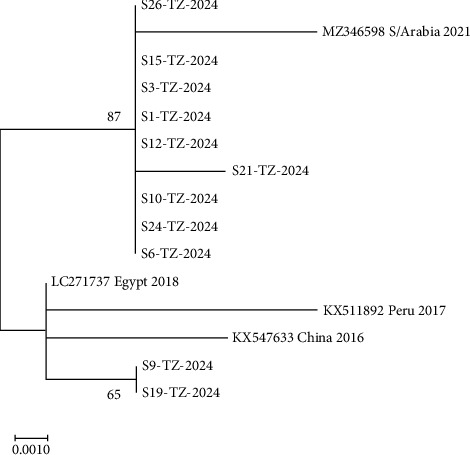
Phylogenetic tree of the COI gene partial nucleotide sequences from this study and selected sequences from GenBank.

Phylogenetic analysis showed that COI gene partial nucleotide sequences S1-TZ-2024, S3-TZ-2024, S6-TZ-2024, S10-TZ-2024, S12-TZ-2024, S15-TZ-2024, S21-TZ-2024, S24-TZ-2024, and S26-TZ-2024 from this study clustered with *Taenia multiceps* from Saudi Arabia (MZ346598) of 2021, while S9-TZ-2024 and S19-TZ-2024 clustered with *Taenia multiceps* from Egypt (LC271757) of 2018, isolate from Peru (KX511892) of 2017, and isolate from China (KX547633) of 2016 during phylogenetic analysis. The phylogenetic tree was constructed using the maximum likelihood method based on the Kimura 2-parameter model using MEGA 7 software (Figure 7).

### 3.5. Association and Risk Factor Quantification Between Cerebral Coenurosis and Household Practices Related to Sheep and Goats

Two variables, namely, the origin of sheep and goats and “communal grazing” (Table 6), were qualified for multivariate logistic regression analysis. In the final multivariate model, “origin of sheep and goats” (Chipogoro: OR = 7.54, 95% CI 1.96–28.97, *p* = 0.003, and Iwondo: OR = 3.90, 95% CI 1.04–14.61, *p* = 0.042) (Table 7) was shown to be linked with cerebral coenurosis, with no confounding variables. The likelihood ratio test generated a Chi-square value of 18.54 with a *p* value of 0.001 from the final developed model.

### 3.6. Knowledge of Small Ruminant's Keepers on Cerebral Coenurosis Disease

The overall average knowledge of cerebral coenurosis among 303 small ruminant keepers was 24.3%. However, the average knowledge on disease detection was 60.7%, while for infection cycle and control, it was 10.85%, and 1.39% was on the zoonotic potential of cerebral coenurosis.

## 4. Discussion

This study established an overall prevalence of 13.5% for cerebral coenurosis in sheep and goats from both postmortem examination of cysts and PCR results. Also, twelve sequenced amplicons verified to be *Taenia multiceps* on BLAST search. In both sheep and goats, the prevalence of the disease was higher in Rudi division, followed by Mima, Mpwapwa, and Kibakwe divisions. The prevalence suggests existence of the disease in the district and contributed to previous studies conducted in Tanzania [5, 12, 18, 21–24].

This prevalence is lower compared to studies conducted in slaughter facilities in Tanzania [18, 22, 24]. This may be due to differences in production systems, eco-climatic conditions, and husbandry practices like routine deworming practices [32–34]. This study provided initial findings for the status of cerebral coenurosis in central Tanzania, emphasizing the need for nationwide control measures. It highlights the importance of focusing on factors that contributed to variations in prevalence rates across regions. This underscores the necessity for policy development to address these regional differences and ensure effective prevention and control measures implementation.

The observed higher prevalence in goats compared to sheep aligns with [3, 24] who established the same in Ethiopia and Tanzania, respectively, but contrary to previous reports [18, 32, 35]. The significant variation in prevalence between sheep and goats may be due to species-specific factors like susceptibility to infection, exposure to the parasite, genetic factors, and grazing habits. Despite the browsing habit of goats, the increased burden of infection may be due to their known curiosity and exploratory nature with the tendency to explore various types of vegetation and possibly soil, hence ingesting tapeworm eggs from contaminated ground material.

The detection of brain cysts of four to 6 cm size in 19.6% of animals presenting neurological signs, with the remained cysts of 2 to 3 cm size found in 90.4% of animals with the absence of neurological signs, provided evidence of the presence of *Coenurus cerebralis* cysts in small ruminants not presenting neurological signs. This is in agreement with the results in the Kiteto District of Manyara region Tanzania [24] and existing literature that the size of the cyst has an impact on interference of the central nervous system and contributes to the presence or absence of neurological signs. Neurological signs occur due to the pressure or inflammation exerted by the cysts on structures of the brain that are more critical for essential brain functions like the thalamus, basal ganglia, and brain stem. The fact that most cysts were recovered in cerebral hemispheres (80.39%) possibly because of the higher biomass of the cerebrum compared to other parts [36], and most of the cysts being few may have also influenced these findings. However, host immunity can also contribute to the presence or absence of neurological signs in affected animals. The observed difference in cyst distribution between cerebral hemispheres may be attributed to factors like differences in vascular supply to the brain hemispheres, variations in the local immune response between the hemispheres, and preference of circulating larval to certain brain entry points. However, more research is needed to assess how these factors contribute to the observed pattern of cyst distribution. Generally, a somehow higher distribution of the cysts to cerebral hemispheres was also reported in studies conducted in Ethiopia [1–3].

Clinical signs observed during ante-mortem inspection related to specific functions of the part of the brain where the cysts were found. Animals with cysts in the left cerebral hemisphere primarily show slight head aversion and circling. Those with cysts in the right cerebral hemisphere and cerebellum predominantly exhibit circling and incoordination.

Furthermore, 29.37% of small ruminant keepers experienced suggestive clinical signs and postmortem features for cerebral coenurosis to their small ruminants over the past 12-month period. This was verified by a reasonable knowledge of the disease detection from small ruminant keepers identified in this study and indicates a significant burden of the disease at the district. Selling was a major option taken by small ruminant keepers when having affected animals and was reported by 44.94% of small ruminant keepers.

The partial nucleotide sequences for the COI gene from this study showed two different clustering patterns based on phylogenetic analysis. One cluster originating from Saudi Arabia in 2021 showed a close genetic relationship with nine sequences from this study. Another cluster, comprised of sequences from Egypt in 2018, Peru in 2017, and China in 2016, included only two sequences from this study. This broader geographic connection implies genetic similarities from different continents, indicating common origins for *Taenia multiceps* in Mpwapwa District.

The study sequences from different divisions within the district revealed minor local variations, and at least one study sequence from each division clustered with a reference sequence from Saudi Arabia. This suggests a potential localized spread, which may be a result of factors like animal movement, trade practices, and local environmental conditions. In Kibakwe and Mpwapwa divisions, all study sequences clustered with reference sequences from the Saudi Arabia cluster. However, Rudi and Mima divisions showed mixed clustering, with two study sequences from Rudi division aligning with the Saudi Arabia cluster and a single study sequence aligning with the Egypt, Peru, and China cluster. Mima Division presented one study sequence aligning with the Saudi Arabian cluster and another aligning with the Egypt, Peru, and China cluster. This implies Rudi and Mima divisions to be a point of multiple sources of *Taenia multiceps*, possibly due to mixed sources of infection or higher movement of animals, which should be considered when developing vaccines or treatments.

An increase in the export of live small ruminants and their products (meat) to Middle East market countries like (Saudi Arabia, Egypt, and China) might have contributed to the spread of the disease to these countries because importation of small ruminants and their products is rarely done in Tanzania.

Multivariate logistic regression analysis identified the source of sheep and goats to be significantly associated with cerebral coenurosis in sheep and goats. These findings are consistent with those identified by [21] in a study conducted in the Arusha region. In the present study, small ruminants from Chipogoro and Iwondo wards were at a higher risk of encountering cerebral coenurosis than those from other wards. This may be a result of weaknesses in interventions in these areas, which may be linked to a lack of knowledge about the disease infection cycle and control, which leads to practices that increase the risk of disease transmission, like giving the brains of affected animals to dogs, throwing away the brains of affected animals with most dogs scavenging, and lack of deworming, or irregular deworming or deworming using non-taeniid drugs.

The knowledge of the disease infection cycle and control and that of zoonotic potential were relatively low, i.e., 10.85% and 1.39%, respectively. Despite this knowledge being relatively low in the current study, it was greater compared to the findings in Iringa and Kiteto districts [23, 24]. This verifies a limited understanding of the disease infection cycle, control, and zoonotic potential among pastoral and agropastoral societies. The average knowledge score on cerebral coenurosis detection was moderate (60.7%), indicating a reasonable understanding of the disease among small ruminant keepers. This level of understanding reflects a significant but not exhaustive understanding of the disease diagnostic procedures, implying the accurate identification and documentation of cerebral coenurosis cases at the district. However, the knowledge on cerebral coenurosis detection obtained in the current study was somehow lower compared to that obtained in Iringa District [23].

### 4.1. Study Limitation

This study experienced minimum funds; hence, only 12 amplicons were selected for sequencing, i.e., three representatives from each division and not all 51 amplicons. Therefore, our prevalence results relied only on postmortem and PCR findings.

## 5. Conclusion

This study established the prevalence of cerebral coenurosis in sheep and goats in Mpwapwa District, with goats exhibiting a higher prevalence compared to sheep. The study found the dominance of the cysts in nonclinically affected animals and cerebral hemispheres of the brain. Again, the study identified two distinct phylogenetic clusters for *Taenia multiceps*: a cluster from Saudi Arabia in 2021 and a broad cluster from Egypt in 2018, Peru in 2017, and China in 2016. Multivariate analysis indicated origin of sheep and goats to be significantly associated with the disease and provided insights into areas to be targeted for control measures. The study also found a relatively low knowledge of small ruminant keepers on the disease infection cycle and control, supported by risky practices like feeding the brains of affected animals to dogs. To mitigate these impacts, educational intervention should be developed and tested before broader dissemination to the livestock keepers to improve their knowledge of disease detection, and avoidance. This educational package should provide culturally appropriate information on the parasite's life cycle and the importance of avoiding practices like feeding brain tissue to dogs and simple disposal methods like burning to minimize the risk. Additionally, strategic deworming programs for dogs particularly in high prevalence areas should be done using praziquantel, every 3 months, to control *Taenia multiceps* and *Echinococcus granulosus* infection, which also pose a significant human health risk. Moreover, the nationwide control plan policy for prevention and control of the disease is to be developed to mitigate the disease impacts. Finally, further research is needed to elucidate factors influencing cyst location within the brain as well as studies in dogs to determine the burden and properly manage the disease infection cycle, as existing studies are limited to northern Tanzania [22, 37] and did not identify the parasite to species level.

## Figures and Tables

**Table 1:** A list of samples selected for sequencing by origin and type of host.

SN	Sample identity	Isolate identity	Host	Division
1	S1-TZ-2024	BC 1	Caprine	Kibakwe
2	S24-TZ-2024	BC 24	Caprine	Kibakwe
3	S26-TZ-2024	BC 26	Caprine	Kibakwe
4	S3-TZ-2024	BC 3	Caprine	Mpwapwa
5	S15-TZ-2024	BC 15	Ovine	Mpwapwa
6	S21-TZ-2024	BC 21	Caprine	Mpwapwa
7	S6-TZ-2024	BC 6	Caprine	Rudi
8	S9-TZ-2024	BC 9	Caprine	Rudi
9	S12-TZ-2024	BC 12	Ovine	Rudi
10	S16-TZ-2024	BC 16	Caprine	Mima
11	S10-TZ-2024	BC 10	Caprine	Mima
12	S19-TZ-2024	BC 19	Caprine	Mima

**Table 2:** Association between cerebral coenurosis and the origin of sheep and goats slaughtered in Mpwapwa District.

Animals examined	Division	Number examined	Number of positive	Chi-square	*p* value
Sheep	Kibakwe	18	0 (0.00%)	5.826	0.115
	Mima	14	1 (7.14%)		
	Mpwapwa	41	1 (2.44%)		
	Rudi	11	2 (18.18%)		

Total		84	4 (4.76%)		

Goats	Kibakwe	43	3 (6.98%)	16.5112	0.0009
	Mima	78	17 (21.79%)		
	Mpwapwa	135	14 (10.37%)		
	Rudi	39	13 (33.33%)		

Total		295	47 (15.93%)		

**Table 3:** Association between cerebral coenurosis and age in sheep and goats slaughtered in Mpwapwa District.

Animal examined	Age (years)	Number examined	Number of positive	Chi-square	*p* value
Sheep	< 2	1	0 (0.00%)	2.0569	0.3576
	2-3	65	2 (3.08%)		
	> 3	18	2 (11.11%)		

Total		84	4 (4.76%)		

Goats	< 2	10	0 (0.00%)	3.1165	0.2105
	2-3	206	31 (15.05%)		
	> 3	79	16 (20.25%)		

Total		295	47 (15.93%)		

**Table 4:** Location of *Coenurus cerebralis* cysts in the brain of affected sheep and goats.

Animal species	Number of examined	Number of positive	Location of cyst in the brain			Chi-square	*p* value
			RCH	LCH	CRL		
Sheep	84	4	2	1	1	52	≤ 0.001
Goats	295	47	24	14	9		
Total	379	51	26	15	10		

Abbreviations: CRL = cerebellum, LCH = left-side cerebral hemisphere, RCH = right-side cerebral hemisphere.

**Table 5:** *Taenia multiceps* COI gene reference nucleotide sequences from BLAST.

Country of origin	Year	Nucleotide identity (%)	Base pairs	Accession number
Egypt	2018	100	447	LC271737
Saudi Arabia	2021	99.51	421	MZ346598
China	2016	99.00	420	KX547633
Peru	2016	98.78	423	KX511892

**Table 6:** Results of univariate analysis of selected household practices and their relationship to prevalence of cerebral coenurosis in small ruminants in Mpwapwa District, Tanzania (*N* = 303).

Variables	*n*	*n*/*N* (%)^1^	Univariate analysis	
			OR (95% CI)	*p* value
Origin				
Kibakwe	93	93/303 (30.7)		
Iwondo	58	58/303 (19.1)	4.55 (1.28–16.20)	0.019⁣^∗^
Chunyu	87	87/303 (28.7)	1.76 (0.49–6.33)	0.381
Chipogoro	65	65/303 (21.5)	8.13 (2.19–30.10)	0.001⁣^∗^
Water source				
Holes and canals				
No	222	222/303 (73.3)		
Yes	81	81/303 (26.7)	0.8 (0.36–1.76)	0.581
Tap water				
No	220	220/303 (72.6)		
Yes	83	83/303 (27.4)	1.20 (0.58–2.51)	0.612
Grazing option				
Communal grazing				
No	21	21/303 (6.9)		
Yes	282	282/303 (93.1)	3.11 (0.41–23.84)	0.245
Cut and carry				
No	286	286/303 (94.4)		
Yes	17	17/303 (5.6)	1.48 (0.41–5.44)	0.546
Deworming				
No	232	232/303 (76.6)		
Yes	71	71/303 (23.4)	0.97(0.44–2.16)	0.955
Owning both small ruminants and dogs				
No	135	135/303 (44.5)		
Yes	168	168/303 (55.5)	1.04 (0.53–2.05)	0.896

*Note: n* = number of small ruminant-keeping households with a particular household practice. *N* = total number of small ruminant-keeping households interviewed. Variables with *p* ≤ 0.25 were included in the multivariate analysis.

^1^Proportion of households which reported a particular household practice.

^∗^ *p* ≤ 0.05.

**Table 7:** Multivariate analysis of possible risk factors in relation to the prevalence of cerebral coenurosis through postmortem examination in Mpwapwa District, Tanzania.

Variables	Categories	*n*/*N* (%)^1^	OR (95% CI)	*p* value
Origin	Kibakwe	93/303 (30.7)		
	Iwondo	58/303 (19.1)	3.90 (1.04–14.61)	0.042⁣^∗^
	Chunyu	87/303 (28.7)	1.41 (0.37–5.41)	0.60
	Chipogoro	65/303 (21.5)	7.54 (1.96–28.97)	0.003⁣^∗∗^

Communal grazing	No	21/303 (6.9)		
	Yes	282/303 (93.1)	2.74 (0.34–21.66)	0.339

*Note: n* = number of small ruminant-keeping households with a particular household practice. *N* = total number of small ruminant-keeping households interviewed.

^1^Proportion of households which reported a particular household practice.

^∗^ and ^∗∗^indicate significant variable categories at *p* ≤ 0.05, but ^∗∗^indicates a variable category with the most lower *p* value between the two, i.e., a more significant variable category.

## Data Availability

The data that support the findings of this study are openly available in GenBank at https://www.ncbi.nlm.nih.gov/genbank/, reference number PP968561 to PP968569.
